# Evaluation of the covariation between leukotriene B4, prostaglandin E2, and hematologic inflammatory parameters in a canine pentylenetetrazole-induced seizure model

**DOI:** 10.3389/fnins.2024.1451902

**Published:** 2024-12-11

**Authors:** Yoonhoi Koo, Taesik Yun, Yeon Chae, Dohee Lee, Hakhyun Kim, Mhan-Pyo Yang, Byeong-Teck Kang

**Affiliations:** ^1^College of Veterinary Medicine, Kyungpook National University, Daegu, Republic of Korea; ^2^Laboratory of Veterinary Internal Medicine, College of Veterinary Medicine, Chungbuk National University, Cheongju, Chungbuk, Republic of Korea

**Keywords:** lymphocyte, albumin, dog, seizure, biomarker

## Abstract

**Background:**

Seizures can cause as well as result from neuroinflammation. This study was performed to identify the hematologic inflammatory parameters (HIPs) and inflammatory mediators that change after a single seizure in a canine pentylenetetrazole (PTZ)-induced seizure model.

**Methods:**

Five healthy Beagle dogs were used in this study. A 3% solution of PTZ was infused until the occurrence of generalized convulsion. Two separate experiments were conducted to observe changes in HIPs over short and long time periods. Blood sampling time points were divided into two periods as follows: short period (baseline, 30, 60, 90, and 120 min after seizure induction) and long period (baseline, 2, 6, 12, 24, and 48 h after seizure induction). The HIPs were calculated, and the serum prostaglandin E2 (PGE2) and leukotriene B4 (LTB4) concentrations were estimated using enzyme-linked immunosorbent assay.

**Results:**

Significant changes (*p* < 0.05) in various HIPs were observed at different time point as follows: neutrophil × monocyte (90 min), neutrophil-to-lymphocyte ratio (60, 90, and 120 min), lymphocyte to monocyte ratio (60 min, 90 min, 120 min, 2 h, 12 h, and 24 h), platelet-to-albumin ratio (90 min), lymphocyte percentage × serum albumin concentration (LA; 60 min, 90 min, 120 min, 2 h), and neutrophil × platelet (6 h). LTB4 concentrations were significantly increased (*p* < 0.05) at 60 and 90 min, and 2, 6, and 48 h after seizure induction. PGE2 was significantly increased only 6 h after seizure induction (*p* < 0.05). LA was one of the HIPs that demonstrated a correlation with LTB4 concentration and showed significant changes that could be observed for a long-period (*p* < 0.05, *r* = −0.4194).

**Conclusion:**

The LA was the only HIP that reflected seizure-associated neuroinflammation. The 5-lipoxygenase pathway might be related to seizure-associated neuroinflammation.

## 1 Introduction

Neuroinflammation, which is a specific type of inflammation occurring in the central nervous system, is attributed to several factors, including infection, traumatic brain injury, toxin, and immune-mediated diseases (Healy et al., 2020; Paudel et al., 2019; Vezzani et al., 2011). The production and activation of cytokines and chemokines are associated with seizure-mediated neuroinflammation (Pracucci et al., 2021; van Vliet et al., 2018). Seizure-mediated neuroinflammation is being studied in veterinary medicine as well as in experimental animals and human patients with epilepsy (Koo et al., 2020; Kostic et al., 2019; Soltani Khaboushan et al., 2022). Seizures can be caused by neuroinflammation or can result in neuroinflammation (Pracucci et al., 2021; Rana and Musto, 2018).

Hematological inflammatory parameters (HIPs) based on circulating blood cell concentration are known to be useful prognostic and potential indicators of the severity of various diseases (Gürsoy et al., 2014; Toptas et al., 2016). Moreover, the neutrophil to lymphocyte ratio (NLR), lymphocyte to monocyte ratio (LMR), and platelet to lymphocyte ratio (PLR) can be considered useful diagnostic markers in patients with epilepsy (Bolayir, 2022; Güneş and Büyükgöl, 2020). Although studies have not investigated HIPs in canine epilepsy, NLR could be used as a diagnostic biomarker in dogs with pneumonia or meningoencephalitis of unknown etiology (Conway et al., 2021; Park et al., 2022).

Studies on inflammatory mediators of epilepsy have investigated novel therapeutic targets of epilepsy or seizures (van Vliet et al., 2018; Vezzani et al., 2019). Novel therapeutic targets of epilepsy include interleukin (IL)-1β, cyclooxygenase (COX), tumor necrosis factor (TNF), and IL-6 receptor (DeSena et al., 2018; Lagarde et al., 2016; Lance et al., 2013). Arachidonic acid can be metabolized by two major enzymatic pathways, including the COX and 5-lipoxygenase (5-LOX) pathways resulting in inflammatory mediators such as prostanoids and leukotrienes, respectively (Julémont et al., 2004). The COX and 5-LOX pathways play major roles in epilepsy-related neuroinflammation, and COX inhibitors and montelukast, which are inhibitors of each pathway, respectively, are known to have anti-epileptic effects (Dhir, 2019; Tesfaye et al., 2021). However, no studies have been conducted on the COX or 5-LOX pathways in canine epilepsy or seizures. Prostaglandin E2 (PGE2) is a representative prostanoid of the COX pathway, whereas leukotriene B4 (LTB4) is a representative leukotriene, which is a product of the 5-LOX pathway (Julémont et al., 2004).

This study aimed to determine the HIPs that change after a single seizure in a canine pentylenetetrazole (PTZ)-induced seizure model, which can be used as a seizure biomarker in dogs. Moreover, an increase in the PGE2 and LTB4 concentrations after a single seizure would help confirm whether the two pathways are involved in seizures in dogs.

## 2 Materials and methods

### 2.1 Animals

This study was approved by the Institutional Animal Care and Use Committee of Chungbuk National University (approval number: CBNUA-1743-22-01). Five healthy Beagle dogs (one male dog and four female dogs) aged between 1 and 2 years old were used in this study. The median body weight of the included dogs was 10.4 (range, 8.9–13.5) kg. All dogs were housed in the laboratory animal care center of Chungbuk National University according to the laboratory animal care and usage guidelines. Temperature and humidity were maintained at 9–25°C and 30–90%, respectively. Artificial lighting was provided for 12 h per day. Toys were provided to each dog for their environmental enrichment. Each dog was fed approximately 20 g/kg of pelleted commercial canine feed (Purina Lab Canine Diet, Purina Mills) twice daily, with water available *ad libitum*.

### 2.2 Canine PTZ-induced seizure model

For seizure induction, 3% PTZ solution in 0.9% NaCl was infused via the vena cephalica antebrachii of dogs as described in previous studies (Löscher et al., 2004; Löscher, 2022). A syringe pump was used for infusion at a rate of 3 mL/min. PTZ solution infusion was continued until the animals exhibited the first generalized myoclonic convulsion. The exact moment of the first generalized myoclonic convulsion was determined by the three observers participating in the experiment. After discontinuation of PTZ administration, 1 mg/kg of diazepam was intravenously administered to prevent seizure exacerbation.

### 2.3 Sample and data collection

Blood samples (3 mL) were obtained from the jugular venipuncture within 5 min at each sampling time point, and the sampling time points were as follows: short period (baseline, 30, 60, 90, and 120 min following seizure induction) and long period (baseline, 2, 6, 12, 24, and 48 h following seizure induction). Complete blood counts (CBCs) were performed for all the obtained blood samples by using ethylene diamine tetraacetic acid-anticoagulated blood on the CBC analyzer (IDEXX ProCyte Dx, IDEXX Laboratories Inc., Westbrook, ME). Serum samples were separated from the obtained blood by centrifugation at 3,500 *g* and 4°C for 10 min within 1 h of sample collection. Serum albumin concentration was measured by a serum chemistry analyzer (IDEXX Catalyst Dx, IDEXX Laboratories Inc., Westbrook, ME). The remaining serum samples were stored at −80°C until enzyme-linked immunosorbent assay (ELISA) was performed.

### 2.4 Calculation of the HIPs

Both the counts and percentage of each type of leukocyte and serum concentration were used for the calculation of the HIPs. HIPs were calculated as follows: multiplication of neutrophil and platelet (NP) = neutrophil counts or percentage × platelet counts, multiplication of monocyte and platelet (MP) = monocyte counts or percentage × platelet counts, multiplication of neutrophil and monocyte (NM) = neutrophil counts or percentage × monocyte counts or percentage, NLR = neutrophil counts/lymphocyte counts, PLR = platelet counts/lymphocyte counts, LMR = lymphocyte counts/monocyte counts, platelet to albumin ratio (PAR) = platelet counts/serum albumin concentration, monocyte to albumin ratio (MAR) = monocyte counts or percentage/serum albumin concentration, and multiplication of lymphocyte and albumin (LA) = lymphocyte counts or percentage × serum albumin concentration (Yamamoto et al., 2021).

### 2.5 PGE2 and LTB4 measurement using ELISA

Serum PGE2 and LTB4 concentrations were estimated using a universal ELISA kit (Elabscience, Wuhan, China) according to the manufacturer’s instructions. The intra-and inter-assay variabilities were both <10%. Optical density was determined at 450 nm using an automated microplate reader (ELx 808, BioTek Instruments Inc., Winooski, Vermont). For comparison with baseline, PGE2 and LTB4 concentrations were calculated as “folds of baseline” due to individual variations.

### 2.6 Statistical analyses

Data were analyzed using a commercial statistical software (Prism 9.4, Graphpad Software Inc., La Jolla, United States). *p*-values were calculated using a two-tailed test. The Kolmogorov–Smirnov test was performed to determine whether the data were normally distributed. To identify the difference from the baseline, the student’s paired *t*-test or the Wilcoxon matched-pairs signed-rank test was performed according to the normality. The Spearman test or the Pearson test was used to identify the correlation between HIPs and inflammatory mediators according to the normality. The Mann–Whitney *U* test or unpaired t-test were used to compare the differences between two groups according to the normality. *p* < 0.05 was considered statistically significant. Normally distributed data are expressed as mean and standard deviation, whereas non-normally distributed data are expressed as median and range.

## 3 Results

### 3.1 Evaluation of the changes in the CBC results following seizure induction

In the short-period experiment, significant reductions of lymphocyte percentage compared to that at baseline (median, 18.8; range, 17.0–32.2; Table 1) were observed at 60 (median, 16.8; range, 15.6–25.9; *p* < 0.05) and 90 (median, 15.7; range, 14.6–21.6; *p* < 0.05) min following seizure induction. In the long-period experiment, a significant increase in the neutrophil counts compared to that at baseline (median, 5.8; range, 4.2–8.5; Table 2) was identified 6 h (median, 8.0, range, 4.5–10.1; *p* < 0.05) following seizure induction, and a significant reduction of lymphocyte percentage compared to that at baseline (median, 19.5; range, 16.3–27.6) was identified 2 h (median, 17.0; range, 9.4–19.0; *p* < 0.05) following seizure induction. No significant change was observed at any time period following seizure induction in the other values.

**Table 1:** Changes in complete blood count results in the short period after seizure induction.

		Baseline	30 min	60 min	90 min	120 min
WBC (counts)	Median	8.8	8.8	8.4	9.1	9.1
	(Range)	(7.5–12.1)	(7.3–14.7)	(7.0–14.7)	(6.8–14.1)	(6.8–14.6)
Neutrophil (counts)	Median	5.3	5.3	5.2	5.4	5.8
	(Range)	(4.7–9.1)	(4.3–11.0)	(4.5–10.9)	(5.0–10.6)	(5.1–11.0)
Neutrophil (percent)	Median	60.8	59.3	62.8	67.7	71.1
	(Range)	(55.8–75.3)	(53.6–75.2)	(53.8–74.5)	(59.6–75.2)	(61.8–75.3)
Lymphocyte (counts)	Median	2.1	2.3	2.2	1.9	1.5
	(Range)	(1.4–3.0)	(1.3–3.0)	(1.2–2.5)	(1.1–2.1)	(1.1–2.2)
Lymphocyte (percent)	Median	18.8	19.7	16.8^*^	15.7^*^	15.5
	(Range)	(17.0–32.2)	(16.0–30.8)	(15.6–25.9)	(14.6–21.6)	(14.4–19.8)
Monocyte (counts)	Median	0.5	0.7	0.7	0.7	0.7
	(Range)	(0.4–0.9)	(0.5–0.9)	(0.5–0.9)	(0.5–1.0)	(0.5–0.9)
Monocyte (percent)	Median	7.0	6.3	6.7	7.3	7.4
	(Range)	(4.1–9.6)	(5.1–9.9)	(5.7–10.9)	(5.6–10.5)	(6.0–9.1)
Platelet (counts)	Median	365	342	327	336	327
	(Range)	(217–722)	(240–705)	(255–672)	(194–636)	(210–605)
Albumin (g/dL)	Median	3.3	3.4	3.4	3.3	3.2
	(Range)	(3.0–3.7)	(3.0–3.7)	(2.9–3.8)	(2.9–3.9)	(2.9–3.9)

*p-*values were calculated using the Student’s paired *t*-test or the Wilcoxon matched-pairs signed-rank test according to the normality. An asterisk indicates a comparison with the baseline. *p < 0.05, significant difference compared to the baseline. WBC, white blood cell.

**Table 2:** Changes in the complete blood count results in the long-period after seizure induction.

		Baseline	2 h	6 h	12 h	24 h	48 h
WBC (counts)	Median	10.1	9.5	10.4	9.9	7.9	9.9
	(Range)	(6.6–12.6)	(8.0–15.7)	(7.1–12.7)	(8.4–11.7)	(6.9–9.7)	(7.8–10.9)
Neutrophil (counts)	Median	5.8	7.4	8.0^*^	7.5	5.0	6.0
	(Range)	(4.2–8.5)	(5.0–12.9)	(4.5–10.1)	(5.2–8.2)	(4.7–7.5)	(4.8–8.4)
Neutrophil (percent)	Median	67.8	71.3	72.6	70.3	68.1	61.2
	(Range)	(57.4–71.6)	(62.4–81.6)	(60.9–80.1)	(61.8–75.3)	(62.6–76.5)	(59.2–76.7)
Lymphocyte (counts)	Median	2.0	1.5	1.6	1.8	1.4	1.9
	(Range)	(1.1–2.8)	(1.3–1.8)	(1.4–1.8)	(1.6–2.1)	(1.2–1.8)	(1.4–2.6)
Lymphocyte (percent)	Median	19.5	17.0^*^	14.6	17.0	19.6	24.8
	(Range)	(16.3–27.6)	(9.4–19.0)	(12.6–25.2)	(16.0–24.0)	(14.7–22.9)	(13.2–27.2)
Monocyte (counts)	Median	0.7	0.7	0.8	0.7	0.7	0.8
	(Range)	(0.4–1.0)	(0.6–1.0)	(0.6–0.9)	(0.6–1.1)	(0.6–0.8)	(0.6–1.1)
Monocyte (percent)	Median	7.7	6.7	7.7	7.6	8.5	9.3
	(Range)	(6.0–8.2)	(6.4–9.6)	(6.2–8.7)	(6.2–9.9)	(6.7–9.6)	(5.9–9.8)
Platelet (counts)	Median	342	354	320	364	381	332
	(Range)	(162–466)	(201–444)	(178–433)	(166–403)	(152–445)	(193–503)
Albumin (g/dL)	Median	3.4	3.3	3.3	3.2	3.2	3.4
	(Range)	(3.2–3.5)	(3.2–3.3)	(3.2–3.6)	(3.1–3.4)	(3.0–3.4)	(3.1–3.6)

*p-*values were calculated using the Student’s paired *t*-test or the Wilcoxon matched-pairs signed-rank test according to the normality. An asterisk indicates a comparison with the baseline. *p < 0.05, significant difference compared to the baseline. WBC, white blood cell.

### 3.2 Evaluation of the changes in the HIP following seizure induction

HIPs were calculated reflecting the percentage and number of each type of blood cells. In the short-period experiment, significant increase in the NLR compared to that at baseline (median, 3.3; range, 1.7–4.4; Table 3) was observed at 60 (median, 3.5; range, 2.1–4.8; *p* < 0.05), 90 (median, 4.2, range, 2.8–5.0; *p* < 0.01), and 120 (median, 4.4; range, 3.1–5.0; *p* < 0.05) min following seizure induction; however, significant reductions of LMR (baseline [median, 3.3; range, 2.6–4.3]; 60 min [median, 2.6; range, 2.4–3.6; *p* < 0.05]; 90 min [median, 2.4; range, 2.0–2.7; *p* < 0.01]; 120 min [median, 2.2; range, 2.0–2.5; *p* < 0.01]) and LA (calculated with percentage; baseline [median, 62.0; range, 54.4–119.1]; 60 min [median, 56.4; range, 48.4–96.9; *p* < 0.05]; 90 min [median, 51.8; range, 42.3–77.6; *p* < 0.05]; 120 min [median, 49.6; range, 41.8–67.3; *p* < 0.05]) were observed at the same time points, respectively. Furthermore, significant reductions of PAR (baseline [median, 107.9; range, 67.8–218.8]; 90 min [median, 101.8; range, 62.6–192.7; *p* < 0.05]) and significant increase in NM (calculated with percentage; baseline [median, 422.3; range, 308.7–535.7]; 90 min [median, 489.1; range, 421.1–625.8; *p* < 0.01]) were observed 90 min following seizure induction.

**Table 3:** Changes in the hematologic inflammatory parameters in the short-period after seizure induction.

		Baseline	30 min	60 min	90 min	120 min
NP (counts)	Median (Range)	1934.5 (1748.0–3934.9)	2005.8 (1341.6–3708.3)	2080.8 (1478.0–3514.6)	2052.5 (1535.7–3192.7)	2199.5 (1532.4–3061.3)
NP (percent)	Median (Range)	20549.5 (16340.1–52633.8)	18501.6 (18048.0–49773.0)	19293.0 (17592.6–49929.6)	20025.6 (14588.8–47127.6)	20208.6 (15813.0–44830.5)
MP (counts)	Median (Range)	259.2 (108.5–310.5)	241.4 (140.4–331.4)	231.2 (176.6–302.4)	241.5 (153.3–322.6)	238.7 (178.7–290.4)
MP (percent)	Median (Range)	2737.5 (889.7–4115.4)	2485.0 (1224.0–4441.5)	2380.0 (1453.5–4300.8)	2590.0 (1086.4–4197.6)	2625.7 (1260.0–4235.0)
NM (counts)	Median (Range)	3.8 (2.3–4.6)	3.8 (1.9–8.1)	4.2 (2.4–9.1)	4.4 (2.3–8.4)	4.5 (2.4–9.7)
NM (percent)	Median (Range)	422.3 (308.7–535.7)	407.4 (367.7–530.6)	439.6 (395.3–586.4)	489.1^**^ (421.1–625.8)	518.7 (451.8–562.4)
NLR	Median (Range)	3.3 (1.7–4.4)	3.3 (1.9–4.7)	3.5^*^ (2.1–4.8)	4.2^**^ (2.8–5.0)	4.4^*^ (3.1–5.0)
PLR (counts)	Median (Range)	139.6 (105.3–512.1)	136.8 (102.6–479.6)	150.0 (111.4–574.4)	188.2 (91.1–600.0)	222.9 (95.9–570.8)
PLR (percent)	Median (Range)	12.8 (11.3–38.4)	15.0 (11.5–35.8)	16.3 (12.6–40.5)	17.6 (12.8–40.5)	20.2 (14.0–39.0)
LMR	Median (Range)	3.3 (2.6–4.3)	3.1 (2.9–4.4)	2.6^*^ (2.4–3.6)	2.4^**^ (2.0–2.7)	2.2^**^ (2.0–2.5)
PAR	Median (Range)	107.9 (67.8–218.8)	103.6 (70.6–207.4)	96.2 (82.3–197.6)	101.8^*^ (62.6–192.7)	96.2 (70.0–189.1)
MAR (counts)	Median (Range)	0.2 (0.1–0.3)	0.2 (0.1–0.3)	0.2 (0.1–0.3)	0.2 (0.1–0.3)	0.2 (0.2–0.3)
MAR (percent)	Median (Range)	2.0 (1.3–2.9)	1.9 (1.5–3.0)	1.9 (1.8–3.2)	2.0 (1.8–3.2)	2.2 (2.0–2.7)
LA (counts)	Median (Range)	6.6 (4.3–11.2)	8.0 (3.9–11.1)	7.1 (3.9–9.4)	6.5 (3.5–7.3)	6.0 (3.4–6.6)
LA (percent)	Median (Range)	62.0 (54.4–119.1)	67.0 (53.4–114.0)	56.4^*^ (48.4–96.9)	51.8^*^ (42.3–77.6)	49.6^*^ (41.8–67.3)

NP, neutrophil × platelet; MP, monocyte × platelet; NM, neutrophil × monocyte; NLR, neutrophil/lymphocyte; PLR, platelet/lymphocyte; LMR, lymphocyte/monocyte; PAR, platelet/albumin; MAR, monocyte/albumin; LA, lymphocyte × albumin. *p-*values were calculated using the Student’s paired *t*-test or the Wilcoxon matched-pairs signed-rank test according to the normality. An asterisk indicates a comparison with the baseline. *p < 0.05, significant difference compared to baseline. **p < 0.01, significant difference compared to baseline.

In the long-period experiment, NP (calculated with counts; baseline [median, 2442.4; range, 675.5–3373.8]; 6 h [median, 2474.2; range, 802.8–3672.3; *p* < 0.05]; Table 4) increased significantly 6 h after seizure induction, whereas LA (calculated with percentage; baseline [median, 63.6; range, 53.8–93.8]; 2 h [median, 56.1; range, 31.0–62.7; *p* < 0.05]) significantly decreased 2 h following seizure induction. Significant decrease in LMR compared to that at baseline (median, 2.8; range, 2.4–3.4) was observed 2 (median, 2.1; range, 1.4–2.5; *p* < 0.01), 12 (median, 2.4; range, 1.7–2.8; *p* < 0.05), and 24 (median, 2.2; range, 2.0–3.0; *p* < 0.05) h following seizure induction.

**Table 4:** Changes in the hematologic inflammatory parameters in the long-period after seizure induction.

		Baseline	2 h	6 h	12 h	24 h	48 h
NP (counts)	Median (Range)	2442.4 (675.5–3373.8)	3116.4 (1272.3–3790.8)	2474.2^*^ (802.8–3572.3)	2268.7 (923.0–3006.9)	1886.0 (746.3–3315.3)	1661.5 (938.0–4215.1)
NP (percent)	Median (Range)	24,487 (9947–33,133)	24,072 (13608–34,721)	19,544 (11303–34,423)	22,495 (10358–30,346)	25,527 (9515–34,043)	20,318 (11426–38,580)
MP (counts)	Median (Range)	283.1 (84.2–380.1)	270.8 (180.9–306.8)	243.2 (97.9–346.0)	254.1 (146.1–311.9)	221.6 (114.0–289.3)	242.4 (150.5–350.7)
MP (percent)	Median (Range)	2,259 (1247–3,755)	2,814 (1929–3,044)	2,684 (1370–3,125)	2,677 (1643–2,766)	2,820 (1459–3,239)	2,967 (1834–3,273)
NM (counts)	Median (Range)	4.8 (1.8–8.5)	5.2 (3.4–13.4)	5.3 (2.5–8.0)	5.0 (3.3–9.3)	3.4 (3.1–4.8)	5.1 (3.5–7.8)
NM (percent)	Median (Range)	472.8 (429.6–535.6)	536.6 (477.7–649.9)	496.6 (489.0–609.8)	512.0 (452.0–681.9)	569.5 (476.1–640.1)	562.4 (452.5–674.2)
NLR	Median (Range)	3.6 (2.1–4.4)	4.2 (3.4–8.7)	5.0 (2.5–6.4)	4.3 (2.6–4.7)	3.5 (2.7–5.2)	2.5 (2.2–5.2)
PLR (counts)	Median (Range)	163.6 (90.5–319.6)	237.3 (112.9–349.6)	179.8 (99.4–318.4)	211.5 (77.6–255.1)	222.2 (84.4–311.2)	171.1 (86.5–303.0)
PLR (percent)	Median (Range)	16.6 (6.1–23.9)	24.7 (10.6–33.4)	19.4 (7.1–33.1)	18.3 (6.9–25.2)	18.6 (6.6–30.3)	13.4 (7.1–33.1)
LMR	Median (Range)	2.8 (2.4–3.4)	2.1^**^ (1.4–2.5)	2.1 (1.7–3.3)	2.4^*^ (1.7–2.8)	2.2^*^ (2.0–3.0)	2.7 (1.4–3.1)
PAR	Median (Range)	103.6 (46.3–145.6)	107.3 (60.9–138.8)	100.0 (53.9–135.3)	113.8 (50.3–130.0)	119.1 (49.0–148.3)	94.9 (56.8–152.4)
MAR (counts)	Median (Range)	0.2 (0.1–0.3)	0.2 (0.2–0.3)	0.2 (0.2–0.3)	0.2 (0.2–0.3)	0.2 (0.2–0.2)	0.2 (0.2–0.3)
MAR (percent)	Median (Range)	2.2 (1.8–2.4)	2.0 (2.0–2.9)	2.3 (1.7–2.7)	2.4 (1.9–3.0)	2.7 (2.2–3.1)	2.7 (1.8–3.2)
LA (counts)	Median (Range)	6.4 (3.5–9.5)	4.9 (4.1–5.9)	5.7 (4.4–5.9)	5.8 (4.9–7.1)	4.6 (4.0–5.6)	6.8 (4.4–9.5)
LA (percent)	Median (Range)	63.6 (53.8–93.8)	56.1^*^ (31.0–62.7)	49.6 (41.9–83.2)	55.4 (49.6–79.2)	65.6 (44.1–71.0)	86.8 (40.9–96.5)

NP, neutrophil × platelet; MP, monocyte × platelet; NM, neutrophil × monocyte; NLR, neutrophil/lymphocyte; PLR, platelet/lymphocyte; LMR, lymphocyte/monocyte; PAR, platelet/albumin; MAR, monocyte/albumin; LA, lymphocyte × albumin. *p-*values were calculated using the Student’s paired *t*-test or the Wilcoxon matched-pairs signed-rank test according to the normality. An asterisk indicates a comparison with the baseline. *p < 0.05, significant difference compared to baseline. An asterisk indicates a comparison with the baseline. **p < 0.01, significant difference compared to baseline.

### 3.3 Evaluation of the changes in the inflammatory mediators following seizure induction

**Figure 1:**
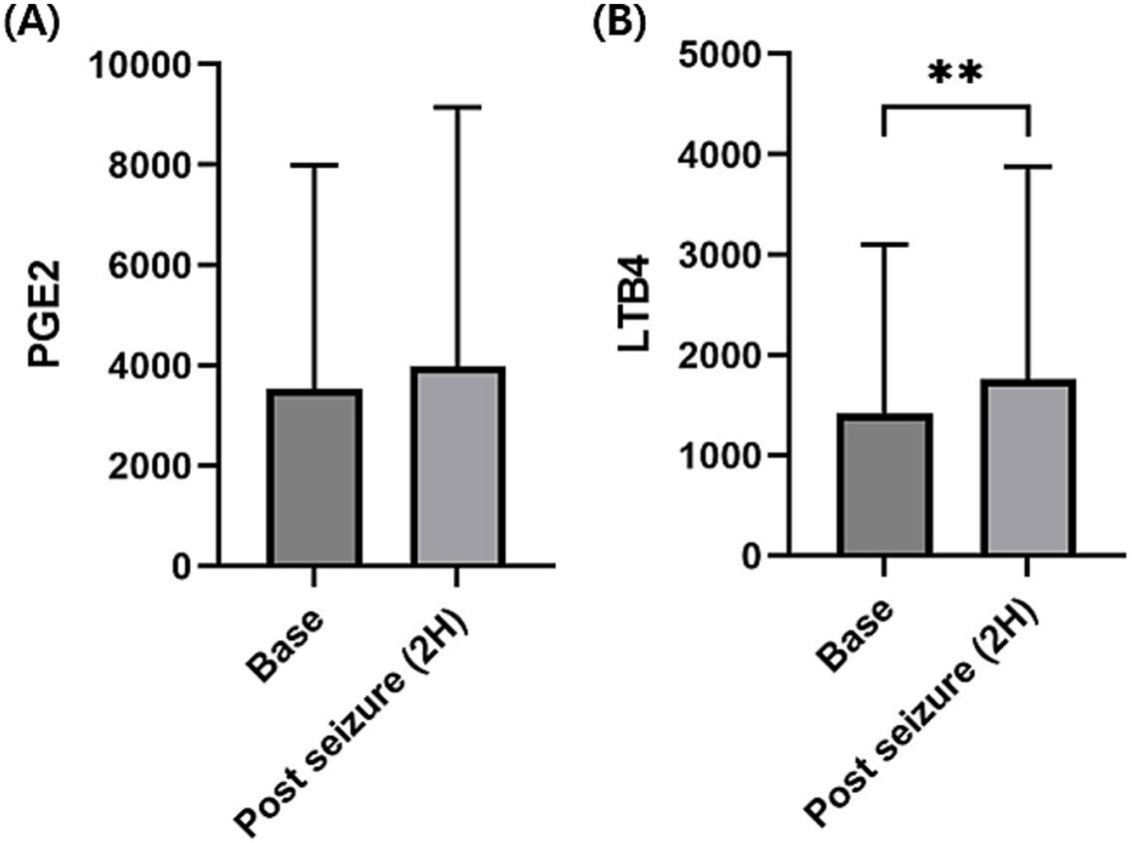
Changes in the serum prostaglandin E2 (PGE2) and leukotriene B4 (LTB4) levels before and 2 h after seizure induction. Serum PGE2 concentrations were not significantly increased at 2 h after seizure induction compared to before seizure induction (base) **(A)**. Serum LTB4 concentration was significantly increased at 2 h after seizure induction compared to before seizure induction **(B)**. *p-*values were calculated using Wilcoxon matched-pairs signed-rank test. An asterisk indicates a comparison with the baseline. **p < 0.01.

Since data at 2 h were available in both the short-and long-period experiment, upon combining these data and comparing the cytokine changes before and 2 h after seizure, PGE2 concentration was not significantly increased (Figure 1A), whereas LTB4 concentration (median, 231.7; range, 56.8–5248.7; *p* < 0.01; Figure 1B) was significantly increased compared to that before seizure (median, 215.5; range, 65.2–3856.1).

**Figure 2:**
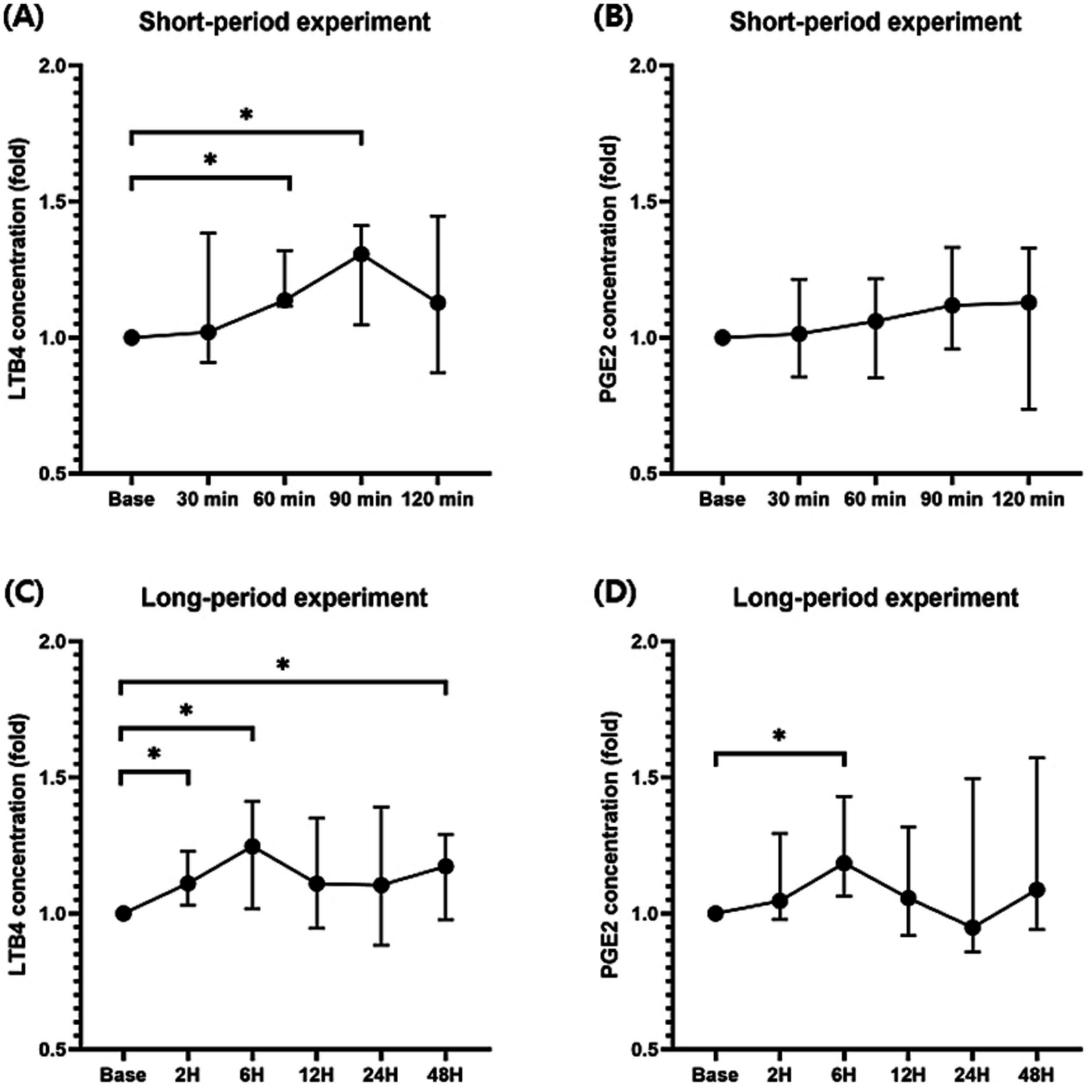
Changes in the prostaglandin E2 (PGE2) and leukotriene B4 (LTB4) levels during the short-and long-period after seizure induction. Significant increases in the LTB4 levels compared to those at baseline were identified 60 and 90 min after seizure induction **(A)**. A significant increase was not identified in the PGE2 levels after seizure induction in the short period **(B)**. LTB4 levels were significantly elevated 2, 6, and 48 h after seizure induction compared to those at baseline before seizure induction **(C)**. A significant increase in the PGE2 levels was observed at 6 h after seizure induction **(D)**. *p-*values were calculated using Student’s paired *t*-test. An asterisk indicates a comparison with the baseline. *p < 0.05.

Because the baseline difference for each individual was clear, the increase in the inflammatory mediators over time was compared based on the fold increase compared to that at baseline. In the short-period experiment, a significant increase in the LTB4 level compared to that at baseline was observed 60 (median, 1.14; range, 1.12–1.32; *p* < 0.05; Figure 2A) and 90 (median, 1.31; range, 1.05–1.41; *p* < 0.05) min following seizure induction, whereas a significant increase was not observed in the PGE2 levels following seizure induction (Figure 2B). In the long-period experiment, LTB4 was significantly elevated at 2 (median, 1.11, range; 1.03–1.23; *p* < 0.05; Figure 2C), 6 (median, 1.25; range, 1.02–1.41; *p* < 0.05), and 48 (median, 1.17; range, 0.98–1.29; *p* < 0.05) h following seizure induction compared to baseline before seizure induction. A significant increase in the PGE2 levels was observed only at 6 (median, 1.19; range, 1.06–1.43; *p* < 0.05; Figure 2D) h following seizure induction.

### 3.4 Correlation between the HIPs and inflammatory mediators

**Figure 3:**
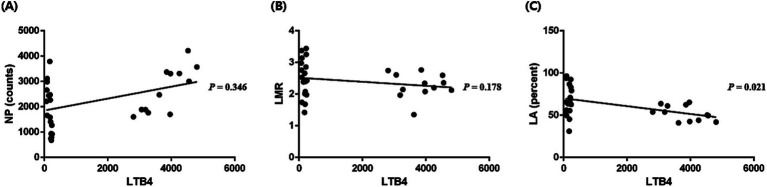
Correlation between the hematologic inflammatory parameters and leukotriene B4 (LTB4) levels. A significant correlation between NP (neutrophil × platelet, calculated with counts) and LTB4 levels was not identified (*p* = 0.346, *r* = 0.18) **(A)**. A significant correlation between LMR (lymphocyte/monocyte) and LTB4 levels was not identified (*p* = 0.178, *r* = −0.25) **(B)**. A significant correlation between LA (lymphocyte × albumin, calculated with percentage) and LTB4 levels was identified (*p* = 0.021, *r* = −0.42) **(C)**. *p-*values were calculated using Spearman test or the Pearson test according to normality.

Correlation was analyzed between the HIPs with significant changes compared to those at baseline. Changes in the LTB4 levels were identified after seizure induction in the long-period experiment. Statistical analysis was performed to confirm the correlation between NP (calculated with counts; *p* = 0.35; *r* = 0.18; Figure 3A), LMR (*p* = 0.18; *r* = −0.25; Figure 3B), and LA (calculated with percentage; *p* < 0.05; *r* = −0.42; Figure 3C) with the LTB4 concentration; only LA (calculated with percentage) correlated with the LTB4 concentration.

## 4 Discussion

This study aimed to investigate the inflammatory mediators, including PGE2 and LTB4, that change after seizures in dogs and determine the HIPs that have a significant correlation with the investigated inflammatory mediators. Inflammatory mediators were investigated to determine novel therapeutic targets of canine epilepsy treatment; HIP was also evaluated in a canine PTZ-induced seizure model to determine the biomarkers for seizure-related neuroinflammation. In the present study, a significant increase in the LTB4 levels following seizure induction and the duration of increase in the LTB4 levels (60 min to 6 h) were identified, suggesting a potential association of LTB4 with seizures. Therefore, the present study suggested that the 5-LOX pathway may be involved in seizure-related neuroinflammation; moreover, the 5-LOX pathway could be considered a novel therapeutic target in canine epilepsy. Furthermore, among the evaluated HIPs, LA, which was identified as the only HIP reflecting the neuroinflammation status associated with LTB4, demonstrated potential to be utilized as a biomarker for seizure-related neuroinflammation.

An increase and decrease in the neutrophil and lymphocyte levels, respectively, are observed following seizures in human patients with epilepsy (Güneş and Büyükgöl, 2020). In addition, several studies conducted on the changes in the circulating T lymphocytes, including CD4+ T lymphocytes and CD8+ T lymphocytes, suggested a possible association between epilepsy and lymphocytes (Bauer et al., 2008; Vieira et al., 2016; Xu et al., 2018). In the present study, an increase in the neutrophil levels following seizure induction was not identified, which was contrary to the findings of a previous study on human epileptic patients; however, reduction in the lymphocyte levels was identified, which was consistent with the findings of the previous study (Güneş and Büyükgöl, 2020). Changes in the leukocytes following seizure reportedly normalize within 2 min in the pilocarpine-induced seizure rat model and after more than 3 days in patients with temporal lobe epilepsy (Bauer et al., 2008; Güneş and Büyükgöl, 2020). In the present study, a significant decrease in the lymphocyte percentage was observed from 60 min to 2 h following seizure induction. Therefore, changes in the leukocytes following seizures in dogs appeared later than that in rats; moreover, the change in the leukocytes was maintained for a shorter duration than that in humans. These differences are suspected to be attributed to interspecies variation.

According to the HIP studies in human epileptic patients, PLR, LMR, and NLR were significantly decreased compared to those in healthy controls (Bolayir, 2022; Güneş and Büyükgöl, 2020). All of these HIPs are directly related to lymphocytes, supporting the association of lymphocytes with epilepsy. In the present study, the HIP identified to be associated with neuroinflammation and seizures was LA, which is also one of the HIPs directly related with lymphocytes. Albumin concentration reportedly increases in the cerebrospinal fluid due to dysfunction of the blood–brain barrier following seizures or status epilepticus; moreover, albumin is released from the blood into the brain tissue due to seizures (Correale et al., 1998; Li et al., 2013). Studies with a larger number of dogs with a long observation period should be conducted to determine the changes in the albumin concentration following seizure in dogs. Although the present study consisted of a relatively short observation period with a small number of dogs, a significant reduction in the LA level following seizures was identified in both the long-and-short period experiments, and a significant reduction in the LA level was observed from 60 min to 2 h following seizure induction. These results suggested that the LA level can be utilized as a canine seizure biomarker.

The COX and 5-LOX pathways play a major role in the pathogenesis of neuroinflammation (Razavi et al., 2021). COX synthesizes prostanoids, including prostacyclin, prostaglandins, and thromboxanes, which are important inflammatory mediators in neuroinflammation (Dhir, 2019). In addition, 5-LOX, one of the key enzymes of neuroinflammation, synthesizes inflammatory mediators, including eicosanoids and leukotrienes (Tesfaye et al., 2021). In the present study, a significant increase in the LTB4 levels was observed over several hours but not for PGE2, which suggested that the 5-LOX pathway that synthesizes LTB4 was activated following seizure induction in dogs. The elevation of inflammatory mediators, including IL-1β, TNF-*α*, and IL-6, was initiated 2 h following seizures and increased to the highest concentration between 6 and 12 h following seizures in the epilepsy mouse model (De Simoni et al., 2000). Furthermore, elevation in the inflammatory mediator levels was maintained for 24 h following seizure induction. Similarly, the expression of IL-1α is also known to occur within 6–8 h of seizure onset, and HMGB1 is significantly increased up to 6 h following the onset of seizures in human epileptic patients (Gahring et al., 1997; Nass et al., 2020). Since the significant increase in the LTB4 concentration in the present study also elevated from 60 min to 6 h, which was similar to the findings of previous studies, it is suspected that several pathways related to neuroinflammation, including the 5-LOX pathway, might be activated simultaneously following seizures. Recently, studies investigating the anti-epileptic effect of the 5-LOX pathway inhibitor, including montelukast and licofelone, in an epilepsy animal model have been published (Razavi et al., 2021; Tesfaye et al., 2021). Moreover, although despite being the only clinical trial, this study demonstrated the anticonvulsant effect of the firocoxib as a COX inhibitor in canine phenobarbital-resistant epilepsy (Fischer et al., 2022). Since the results of the present study suggest the possible anti-epileptic effect of the 5-LOX pathway inhibitor, further studies are warranted to confirm the anti-epileptic effect of 5-LOX inhibitors in an epilepsy dog model or canine epilepsy patients.

To determine a precise seizure monitoring marker reflecting the neuroinflammation status, which was one of the main objectives of this study, the discovered biomarker should satisfy the following two conditions: maintain changes over a long period of time and correlate with inflammatory mediators. In the present study, only LA was identified as an HIP that satisfied both conditions; therefore, LA can be utilized as a biomarker for monitoring neuroinflammation and seizures in dogs. To investigate LA as a diagnostic or screening marker for canine epilepsy, additional studies in canine epilepsy patients, not in dogs with single seizure induction as in this study, are warranted.

This study had several limitations. First, the sample size was small, consisting of only five dogs from a single breed, which may have contributed to the negative findings. Future studies with a larger sample size with variety of breeds should be conducted to provide more comprehensive results. Second, electroencephalography (EEG) was not performed due to excessive movement of the dogs. General anesthesia using isoflurane or sedative agent administration, including medetomidine, was considered to control excessive movement; however, isoflurane possesses anti-epileptic effects, and several sedative agents are known to affect the results of EEG (Itamoto et al., 2002; Langmoen et al., 1992). Thus, EEG was not performed in the present study. Therefore, more deteriorated neuroinflammation status could have been recorded because the onset of seizure was identified only by visual evaluation, which could be recorded later than the actual occurrence of seizures. Third, HIPs or neuroinflammatory mediators could be affected by the diazepam administered to prevent exacerbation of seizures. Although diazepam did not affect lymphocyte proliferation in an *in vitro* experiment using mice, no study has been conducted in dogs (de Lima et al., 2010). Fourth, as measurement of neuroinflammatory mediators except PGE2 and LTB4 were not performed, accurate identification of association between neuroinflammation and seizures was not possible. Additionally, other leukotrienes associated with the 5-LOX pathway were also not measured. Therefore, further studies including measurement of other leukotrienes and neuroinflammatory mediators are needed. Fifth, since the analysis was performed only for limited time points following seizure induction, the peak times or half-lives of all the HIPs and inflammatory mediators could not be identified. Therefore, accurate biological information is warranted to enable the use of LA as a biomarker. Thus, more detailed dynamic studies including identifying changes in inflammatory parameters within the first 30 min after seizure induction should be conducted.

## 5 Conclusion

Since a significant increase in LTB4 concentration was observed following seizure induction, the 5-LOX pathway was presumed to be associated with seizure-related neuroinflammation in dogs. Therefore, the 5-LOX pathway could be considered a novel therapeutic target for managing seizure-related neuroinflammation in dogs. Furthermore, LA was determined as the only HIP that reliably reflects seizure-related neuroinflammation, positioning it as a potential biomarker for canine epilepsy.

## Data Availability

The original contributions presented in the study are included in the article/supplementary material, further inquiries can be directed to the corresponding author.
